# Supplementary material for: Are carbon emissions trading and green financial instruments synergistic? -Comprehensive quantitative research based on content analysis
Source: PLoS One. 2024 Mar 7;19(3):e0298601. doi: 10.1371/journal.pone.0298601 (PMC10919601; doi:10.1371/journal.pone.0298601)
Supplement: S1 Data — (ZIP) [file pone.0298601.s001.zip › Data/地级市Z_F工作报告.zip!/║■▒▒/╗╞╕╘╩╨/└┤╫╘╣1⁄2╓┌║┼í╛┬φ┐╦╩2╛▌═°í┐_2012.docx]

各位代表：

现在，我代表市人民政府向大会报告政府工作，请予审议，并请市政协各位委员和其他列席人员提出意见。

一、过去五年工作回顾

本届政府的五年，是黄冈发展历程中极不平凡的五年，是我市改革开放和现代化建设取得重大成就的五年。五年来，我们在市委的坚强领导下，深入贯彻落实科学发展观，紧紧抓住建设武汉城市圈“两型社会”综合配套改革试验区、大别山革命老区经济社会发展试验区等重大机遇，有效应对国际金融危机的巨大冲击，战胜了旱涝雨雪冰冻严重自然灾害，同心同德、克难奋进，较好地完成了市三届人大一次会议确定的目标任务，黄冈面貌发生了新的历史性变化，谱写了科学发展的新篇章。

经济实力明显提升。经济总量快速增长，预计2011年全市地区生产总值达到1012亿元，年均增长14.5%。全社会固定资产投资821亿元，年均增长34%。规模以上工业增加值240亿元，年均增长32.7%。社会消费品零售总额477亿元，年均增长21.1%。全口径财政收入133.3亿元，年均增长31.1%，其中地方一般预算收入51.9亿元，年均增长27.6%。金融机构存贷款余额分别达到1158亿元和465亿元，年均增长21.1%和15.6%。工业经济迅猛发展，通过搭建地方政府投融资平台累计融资200多亿元，建成各类开发区和工业园区34个，引进项目4577个，其中亿元以上项目349个；初步形成产业集群31个，其中7个进入全省重点行列。五大支柱产业年总产值都突破了100亿元，新增亿元以上企业172家。高新技术产业增加值超过60亿元，年均增长33.2%。建筑业综合实力位居全省第二，3家企业名列全省20强。农业基础地位不断夯实，粮食增产4.3亿公斤，达到32.7亿公斤，实现“八连增”，油料、畜禽、水产、板栗、茶叶等主要农产品全面增长。农业产业化经营深入推进，新增市级以上龙头企业136家，组建农民专业合作社1756家，土地流转面积61万亩，农机化综合水平达到58%。建成全省最大的奶牛和小龙虾养殖基地，获得国家地理标志保护产品24个，居全国地级市之首。服务业发展活力增强，旅游业进入规模发展阶段，建成4A级景区8个，年接待游客近1000万人次。商贸、物流、金融、保险、通信、石油、烟草、房地产等行业快速兴起。区域经济竞相发展，市区主要经济指标增幅高于全市平均水平；县域经济在激励竞争中加快成长，6个县市区生产总值过100亿元，6个县市财政收入过10亿元。

城乡面貌明显改观。五年累计完成投资2739.8亿元，年均增长37.5%。交通建设，完成投资257.8亿元，新增通车里程7765公里，其中高速公路新增336公里，在全省率先实现县县通高速。总投资14亿元的大别山红色旅游公路全线通车。黄鄂高速公路延长线和麻城至武穴高速公路正式动工。黄冈长江大桥、武汉至黄冈城际铁路和黄鄂高速公路将于2013年同步建成通车。黄冈与武汉同城化步伐加快，区位交通优势进一步增强。水利建设，完成投资55.1亿元，整险加固长江回水堤防57公里、大中型水库106座、小Ⅰ型水库119座，治理水土流失面积1170平方公里。城镇建设，市区完成投资33亿元，编制实施了土地利用总体规划、城市总体规划和控制性详细规划、城东新区规划以及一批重大专项规划，主次街道刷黑工程基本完成，一批社会事业工程项目建设顺利推进。遗爱湖公园完成投资超过5亿元，成为提升黄冈城市品位的标志性工程。持续开展“四城联创”、“城管会战”和网格化管理，成功创建省级卫生城、省级文明城和省级园林城。小城镇建设和社区“八个一”工程全面实施，城镇化水平提高了5.1个百分点。新农村建设全面推进，累计硬化通村公路1.4万公里，油路（水泥路）、固定电话和有线电视实现村村通，客车、宽带通村率分别达到95%和92.5%。能源建设，大别山电厂、白莲河抽水蓄能电站、鄂东500千伏环网和天然气工程建成投产。农村清洁能源入户28.3万户。生态建设，实施长江生态文明示范带和农村环境连片整治示范工程，建成污水处理厂13座、垃圾处理场11个，依法关闭高能耗高污染企业110家，圆满完成了节能减排任务。林业重点工程建设投入超过20亿元，森林覆盖率达到43%。争取国家和省土地整理资金25.5亿元，建成了一批高产农田。

改革开放明显推进。启动并推进大别山试验区建设，创建了多元化投入机制、科技创新机制、人才支撑机制和环境保护与生态补偿机制，与74个省直部门建立了战略合作关系，32个“点中点”完成投资43.5亿元。编制并实施了“两型社会”建设规划和长江经济带开发规划，长江岸线资源管理与利用得到加强，港口、码头和重点产业建设加快推进。着眼于破解经济社会发展资金瓶颈制约，组建和壮大了城投公司、经发投公司、大别山旅游投资公司和中小企业担保公司，强力推动企业上市，组建农村合作银行，引进股份制金融机构，运用BT、BOT等市场融资模式，建设了一大批基础设施项目和社会事业项目。农村公益性服务“以钱养事”新机制基本建立，集体林权制度改革验收达标。新一轮市县政府机构改革基本完成，事业单位改革取得初步成效。招投标综合监管体系不断完善。价格和粮食流通体制改革取得新的进展，粮油收储加工能力大幅提升。加强国有资产监管，切实履行出资人职责。财税征管改革不断深化，部门预算、财政与编制政务公开全面推行。毫不动摇地支持民营经济发展，个体工商户发展到21.3万户，私营企业发展到1.4万户。累计实现外贸出口17.1亿美元，直接利用外资5.7亿美元。成功举办了全国基础教育·黄冈论坛、首届中国·黄冈大别山旅游节、第六届第七届湖北省黄梅戏艺术节和全市第二届第三届运动会等重大节会活动。

人民群众实惠明显增多。连续五年坚持每年为民办8—10件实事，解决了一批人民群众最关心、最直接、最现实的利益问题。城乡居民收入较快增长，预计2011年城镇居民人均可支配收入14659元，年均增长16%；农民人均纯收入5375元，年均增长13.3%。就业和社会保障水平稳步提高，城镇就业累计增加26.8万人，帮助就业困难人员和零就业家庭实现就业12.2万人次，转移农村劳动力85.3万人次，就业培训160.4万人次，城镇登记失业率控制在4.4%以内。各项社会保障制度覆盖面逐步扩大，新型农村合作医疗参合率达到98.9%，职工养老保险实现省级统筹，医疗、工伤保险实现市级统筹，城乡居民养老保险基本实现了全覆盖。城镇住房公积金制度建设加强。保障性住房建设投入5.3亿元，建成面积71.4万平方米。社会救助、灾民安置等制度不断完善。实施整村推进、产业扶贫、搬迁扶贫和老区建设，72万低收入人口摆脱了贫困。

各项社会事业明显进步。文化建设迈出重要步伐。累计投入10多亿元，建成了一批重点文化惠民工程，农村和城市社区文化设施建设不断加强。出台了一系列加强名人文化建设和文物保护的政策措施，支持创作了一批文化艺术精品。电影《黎明行动》荣获全国“五个一工程”奖，广播剧《天堂岛》获全国金奖，黄梅戏《李四光》、《东坡》在人民大会堂和国家大剧院成功演出。倾力打造教育品牌，优化教育布局，深化教学改革，落实助学帮困政策，推进校舍安全工程和农村合格学校建设，促进了各类教育持续协调发展。实施人才强市战略，推进科技改革创新，完成科技计划项目397项，获省市科技进步奖173项，新认定高新技术企业28家。蝉联两届“全国科技进步先进市”。大力实施医药卫生体制改革，落实国家基本药物制度，加强医疗卫生服务体系建设，城乡“1560就医圈”基本形成。免费为4300名贫困白内障患者实施了复明手术，被授予“全国白内障无障碍市”。着力开展出生人口性别比偏高专项治理，低生育水平保持稳定，计生服务体系建设全面加强。荣获“全国群众体育先进市”称号。扎实开展放心早餐工程建设和食品药品安全监管，加强城区猪肉、蔬菜等市场管理，“六大食品安全”工程建设做法在全省推广。设立大别山质量奖，精品名牌战略取得可喜成果。全面落实安全生产责任制，加强重点领域安全整治和监管，深入开展隐患排查和打非治违活动，有效防范了重特大安全事故的发生。组建了综合应急救援队伍。加强了消防安全工作。四川灾区援建、对口援疆和南水北调工程移民试点安置工作，得到国务院、省政府的充分肯定。统计、调查、人防、气象、防震、民族、宗教、方志、档案、保密、经研、对台、侨务等工作得到加强，妇女儿童、残疾人、老龄事业健康发展。

精神文明和民主法制建设明显加强。扎实推进社会主义核心价值体系建设，群众性精神文明创建活动不断深入，社会文明程度进一步提高。民主建设有序推进，自觉接受市人大的法律监督、工作监督和市政协的民主监督，认真听取人大代表、政协委员的意见和建议，五年办复人大代表建议597件、政协提案1292件，办结率和满意率不断提高。尤其是集中力量办理了公路治超、农村建房规划与用地管理、社区建设、市区学校布局调整等四项人大议案，探索了成功经验，取得了良好成效。广泛听取民主党派、工商联、无党派人士的意见和建议，密切联系工会、共青团、妇联、科协等人民团体。加强服务型政府建设，强化普法和政府法制工作，倡导“八简两风”，深入开展“治庸问责”和“三万”活动，在全省率先推行公务员量化考核，机关效能和执行力不断提高。政务公开和电子政务建设全面推进。加强廉政风险预警防控，严格“三公”经费管理，扎实开展经济责任审计，深入开展治理商业贿赂和工程建设领域突出问题等专项行动，廉政勤政建设取得新成绩。深入推进法治黄冈、平安黄冈建设，完善社会治安防控体系，“打黑除恶”、“扫黄打非”、破案追逃、反抢反盗等严打整治行动成效显著。所有县市区都被命名为平安县市区，“法务前沿工程”得到中央领导的充分肯定。

全民国防意识不断增强，国防动员建设取得新成效。驻黄部队和民兵在支持地方经济建设、遂行急难险重任务中作出了重大贡献。发扬拥军优属、拥政爱民优良传统，荣获“全国双拥模范城”。

刚刚过去的2011年，面对复杂严峻的国内外经济形势和旱涝急转的严重自然灾害，我们团结带领全市人民顽强拼搏，锐意进取，保持了经济平稳较快增长，实现了“十二五”良好开局。预计全市地区生产总值增长13.5%，规模以上工业增加值增长23%，全社会固定资产投资增长27.2%，社会消费品零售总额增长17%，外贸出口增长15%，地方一般预算收入增长17.8%，城镇居民人均可支配收入增长14.2%，农民人均纯收入增长16%。

过去五年，我市经济社会发展取得的巨大成就，得益于省委、省政府和市委的正确领导，得益于全市人民的团结奋斗，得益于社会各界的大力支持。在此，我谨代表市人民政府，向辛勤工作在各行各业的全体市民，向给予政府工作大力支持的人大代表和政协委员，向各民主党派、工商联和社会各界人士，向驻黄部队和武警官兵，向所有关心支持黄冈改革发展的同志们、朋友们，表示崇高的敬意和衷心的感谢！

在总结成绩的同时，我们更清醒地认识到前进的道路上还面临不少困难和问题：经济总量不大、市场主体不多、产业结构不优；工业集群化、农业产业化、服务业现代化和新型城镇化程度不高，经济增长的基础不牢；资源环境约束日益突出，经济结构优化升级任务艰巨；社会事业发展欠账较多，社会管理还有许多不适应的地方；投资创业环境仍需改善，政务服务能力有待进一步提高。我们一定要高度重视这些困难和问题，采取有力措施，认真加以解决。

二、今后五年总体要求

今后五年是我市全面建设小康社会的关键时期。面对前所未有的机遇，面对前所未有的挑战，我们必须增强时不我待的历史责任感和现实紧迫感，立足全市人民苦干实干形成的良好发展基础，珍惜全市上下团结进取的大好局面，不辜负全市人民加快富民强市的迫切愿望，振奋精神，抢抓机遇，开拓创新，努力开创跨越式发展的新局面。

今后五年要围绕全面完成“十二五”规划，衔接启动“十三五”规划，牢牢把握政府工作的总体要求：坚持以邓小平理论和“三个代表”重要思想为指导，深入贯彻落实科学发展观，按照市第四次党代会的决策部署，围绕实现“三个跨越”、“三个崛起”、“三个提升”的奋斗目标，以转变经济发展方式为主线，以改革开放为动力，以保障和改善民生为出发点和落脚点，全面实施开创战略，大力推进新型工业化、新型城镇化、农业现代化，全力打造红色黄冈、绿色黄冈、发展黄冈、富裕黄冈，奋力推进跨越发展，为建设富裕文明新黄冈而努力奋斗。

实施开创战略、推进跨越发展，是市第四次党代会对未来五年全市经济社会发展的战略决策，是新一届市政府工作的主旋律。开创战略是开放先导、创新驱动、“一区两带”、试验跨越的集合体。开放先导是方向、创新驱动是动力、“一区两带”是载体、试验跨越是路径，既各有侧重，又辩证统一，相互渗透、相互依存、相互促进、相辅相成，统一于推进黄冈跨越发展的具体实践之中。全面实施开创战略，奋力推进跨越发展，必须切实做到：

——加快转变发展方式和提升综合实力。做大做实做强工业、现代农业、现代服务业和“三驾马车”，促进经济增长向三次产业协同带动、消费投资出口协调拉动、内生型外向型协力驱动转变。力争到2016年，地区生产总值突破2000亿元，规模以上工业增加值突破600亿元，全口径财政收入突破200亿元，地方一般预算收入突破100亿元。坚持工业强市不动摇，以优化产业结构为重点，以实施百亿园区、百亿产业、百亿企业“三个一百”工程为载体，大力发展节能环保、新能源、新材料、电子信息、生物工程等高新技术产业，发展壮大钢构、造船、窑炉、林纸、光热等产业，改造提升医药化工、纺织服装、汽车配件、建筑建材、机械、食品等产业。着力培植一批产值过200亿元的支柱产业，力争医药化工、食品饮料、建筑建材三个产业产值达到500亿元，建成一批销售收入过10亿元、50亿元、100亿元的骨干企业，新增3—5家上市企业。实施农业强市战略，重点抓好龙头企业，大力构建现代农业生产体系、农业产业化经营体系、农业社会化服务体系、农产品质量安全体系、农业科技支撑体系、农业生态环境保护体系，培育一批带动力强的龙头加工企业，建设一批种植业板块、畜牧业小区和水产业片带，创建一批农产品加工大县，培植一批农产品知名品牌。把发展现代服务业作为转变发展方式的战略重点，着力发展金融保险、现代物流、信息服务、科技咨询、研发设计、服务外包等产业。加快培育旅游支柱产业，全面推进旅游经济强市建设。把扩大投资、推进项目建设作为经济工作的第一抓手，坚持直接融资与间接融资、引进外资与吸引内资、争取政策性投资与激活民间投资相结合，力争全社会固定资产投资年均增长25%以上。重点完善高速公路网、快速干线交通网、农村公路网、低碳水运网和综合交通运输网，努力建设“15分钟快车道”、“90分钟交通圈”。加强农田水利建设，加固沿江沿湖圩堤，整治病险水库，推进中小河流治理，完善排涝抗旱设施，提高防灾抗灾减灾能力。加强能源建设，加快推进核能、水能、风能、太阳能、地热能和生物质能等重点项目建设。把增加投资与扩大消费、出口更好地结合起来，改善消费环境，培育消费热点，扩大消费需求；抓好出口基地建设，扶持壮大一批出口龙头企业，保持对外贸易稳定增长。

——构建战略定位明确、特色优势鲜明的区域发展格局。加快黄冈临港经济区建设。按照“一港一线一城多园”的发展思路，主动对接大武汉、连通大武汉、融入大武汉，着力构建临港产业、综合交通、综合物流三大体系，加快形成与武汉同城化发展新格局。坚持高昂市区龙头，深入推进市区一体开发，打造现代产业支撑体系，壮大市区综合实力。力争五年建成百亿工业园区3个，黄冈经济开发区力争跻身国家级开发区。加快城东新区建设，推进城区“东扩北进、跨河发展”，抓紧完成行政中心东迁、市政设施、“一河两湖”水生态修复、跨河通道、城际铁路站区、文化教育医疗等重点项目建设任务，推进旧城改造、赤壁风景区保护与开发、遗爱湖综合治理、江滩整治以及沿江风光带建设。加强城市规划建设管理，开展新一轮“四城联创”，积极创建国家级园林城市、卫生城市、文明城市和省级环保模范城市。力争到2016年城东新区初具雏形，市区建成区面积达到60平方公里，人口达到50万人。提升县城规划、建设、经营和管理水平，抓好重点镇、口子镇建设，促进土地节约集约利用。加强农村道路、通信、广电、安全饮水、电力等基础设施，整体推进新农村建设。坚持生态立市，进一步加强生态保护，提高森林覆盖率，改善人居环境，确保老百姓喝上干净水、呼吸上新鲜空气、吃上放心食品。加快推进长江经济带建设。着力抓好沿江主要港口、航道及配套交通基础设施建设，构建沿江综合交通运输体系和沿江产业密集带。加强与周边城市协作，推进跨江联合开发。加快推进大别山旅游经济带建设。以旅游资源整合和区域合作为抓手，以核心景区为重点，大力实施旅游开发、生态建设和环境保护，努力建成国内一流、世界知名的生态文化旅游目的地。坚持新型工业化、新型城镇化与农业现代化“三化融合”，促进区带互动、两带联动，形成区域经济发展的新格局。力争有若干县市区进入全省县域经济第一方阵。

——建设创新型城市。围绕体制机制创新，大力推进重点领域和关键环节改革，培育壮大市场主体，扶持发展非公有制经济，充分发挥市场配置资源的基础性作用，基本形成有利于科学发展、跨越发展的体制机制。围绕科技创新，深入实施自主创新和人才强市战略，构建以企业为主体、市场为导向、产学研结合的开放型区域创新体系，建设高新技术产业园和留学生创业园；加强引才引智和国内外人才交流，构建多层次人才培养体系。围绕文化传承创新，全力建设文化强市。大力发展公益性文化事业，全面实施名人文化和文物事业发展规划，加强文化遗产保护，打造“中国名人之都”；加大公共文化事业投入，建成黄梅戏大剧院、市图书馆、市档案馆、市妇女儿童中心等重点文化设施，构建城区“10分钟文化圈”、农村“10里文化圈”，推出一批文化精品力作；充分挖掘文化资源，着力打造红色文化、名人文化、戏曲文化、东坡文化、禅宗文化、健康养生文化等特色文化品牌，建设一批重点企业和文化园区，壮大“一县一品”文化产业。优化教育资源配置，提高教育教学质量，提升黄冈教育品牌影响力和竞争力，努力实现教育大市向教育强市跨越。围绕社会管理创新，完善公共服务、公共安全和社会组织管理体系，基本实现管理网络化、服务全程化、覆盖全域化，维护社会和谐稳定。围绕工作创新，强化“能力席位”意识和“一切取决于自己”的理念，完善考核机制，建立倒逼机制，引进竞争机制，创造性地推动各项工作落实。

——探索试验跨越的新路径。建设武汉城市圈“两型社会”综合配套改革试验区和大别山革命老区经济社会发展试验区，是中央和省委、省政府对黄冈的亲切关怀与殷切期待，是我市最大的发展机遇和“金字招牌”，必须大力弘扬革命老区敢想敢干、敢为人先的优良传统，用足用活政策，大胆改革试验。坚持用试验跨越的视野，寻找黄冈在全球、全国、全省经济分工与发展中的定位，更加积极主动地实施开放先导战略，敢开放、真开放、全开放，以开放促改革、促创新、促发展。坚持用试验跨越的思维，正确认识和妥善处理经济社会发展的重大关系，把握全局，统筹谋划，发挥优势，突出特色，分步推进，重点突破。统筹经济与社会发展关系，坚持以经济建设为中心，全面推进政治建设、文化建设、社会建设和生态文明建设。统筹当前发展与长远发展关系，在谋划长远发展中立足当前发展，在抓好当前发展中为长远发展创造有利条件。统筹处理政府与市场的关系，在合理有效配置资源中，更好地发挥市场基础性作用和政府调控作用。坚持用试验跨越的办法，健全改善民生的长效机制，让人民群众更好地享受发展成果。力争到2016年，城镇居民人均可支配收入达到2.6万元，农民人均纯收入达到1万元以上；困难群众基本生活保障制度、城乡居民基本医疗保障制度、城乡居民基本养老保险制度实现全覆盖。

三、高标准做好2012年各项工作

今年是实施“十二五”规划承上启下重要的一年。全市经济社会发展主要预期目标是：地区生产总值增长13%左右；规模以上工业增加值增长20%；全社会固定资产投资增长25%；社会消费品零售总额增长17%；外贸出口增长10%以上，实际利用外资增长8%以上；地方一般预算收入增长15%；节能减排完成省下达指标；城镇居民人均可支配收入和农民人均纯收入分别增长12%；城镇登记失业率控制在4.6%以内；居民消费价格指数涨幅控制在4.5%左右；人口自然增长率控制在6‰以内。

实现上述目标，要坚持稳中求进、紧中求活，把稳增长、控物价、调结构、惠民生、抓改革、促和谐更好地结合起来，重点做好以下八个方面的工作。

（一）努力扩大投资和消费需求

确保投资较快增长。认真贯彻国家宏观调控政策，调整优化投资结构，提高投资效益，集中建设一批影响全局、支撑长远发展的大项目。突出抓好续建和新开工的亿元重点项目，特别是列入全省重点建设计划的林浆纸一体化、黄冈长江大桥、武汉至黄冈城际铁路、黄鄂高速、麻竹高速、麻城至武穴高速等重大项目建设，扎实推进浠水核电、抽水蓄能电站、风力发电、黄冈电厂、大别山电厂二期工程、黄冈机场等重大项目前期工作。增强项目谋划的针对性和有效性，力争有一批项目列入国家和省投资计划，确保我市争取中央和省的项目资金稳中有升。

提高招商引资质效。按照“开展大招商，承接大转移，引进大项目，建设大园区”的思路，整合招商力量，形成招商合力。加强与央企的合作对接，与发达地区对接，与大型民营企业对接，与国内外大公司对接。支持知名企业在黄冈设立区域性总部和研发营销中心。加强项目落地跟踪服务，确保项目尽快建设，尽快竣工投产。

加强生产要素保障。建立金融机构信贷目标考核和激励竞争机制，确保“三农”、中小企业和县域经济的贷款增量不低于上年实绩、不低于存款增幅。把融资担保体系建设作为培育市场主体、支持企业发展的重要平台，进一步壮大融资担保公司的实力。积极引进股份制银行、创投公司和信托公司，争取一批新的农村合作银行、村镇银行和小额贷款公司投入运营。加快企业上市步伐，综合运用各种金融工具，扩大资本市场规模。有效推进农村金融服务全覆盖，进一步优化金融生态环境。以城乡建设用地增减挂钩试点为抓手，以低丘缓坡地综合开发利用为平台，整合资源，向内挖潜，强化经营管理，保障重点项目用地需求。加强煤电油气运综合协调，突出抓好电力基础设施建设和成品油市场供给，满足经济发展需要。

进一步扩大消费需求。落实消费政策，培育消费热点，增加廉租住房和普通商品住房供应，促进房地产业健康发展。积极发展城市社区商业，大力改善农村消费环境，继续推进“万村千乡”市场工程和新农村现代流通网络工程。深化收入分配制度改革，提升居民消费水平。深入整顿和规范市场秩序，继续实施食品安全工程，不断提高食品、药品、餐饮卫生安全监管水平。严厉打击侵犯知识产权和制售假冒伪劣商品行为。采取综合措施，保持物价总水平基本稳定。

加快发展现代服务业。深入开展中国优秀旅游目的地创建活动，积极培育壮大旅游市场主体，加强旅游星级饭店建设，加大特色旅游产品和旅游商品开发力度，促进旅游业各要素协调发展。加快发展以现代物流、信息、科技服务、中介服务等为重点的生产性服务业，培育大型物流企业。支持发展商业保险。抓好社区生活服务业。

（二）大力推进新型工业化

加快园区建设。把建设大园区作为聚集大产业、培育大企业的主要载体，进一步加快工业园区建设步伐。按照布局集中、土地集约、产业聚集、生态环保的原则，创新园区建设运营管理体制机制。采取政府引导、市场运作、社会参与等多种方式，灵活运用BT、BOT等筹融资办法，建设一批特色产业园区。将工业化与城镇化紧密结合起来，在加快水、电、路、通信等基础设施建设的同时，着力推进园区政务、商务、服务三配套，以标准化、规范化、功能化的园区，吸引客商落户，承接产业转移，加快工业经济发展。

促进产业集聚。坚持市场导向，发挥龙头企业在产业集群中的辐射带动作用，注重引进上下游配套项目，延长产业链条，扩大产业规模。加速壮大食品饮料、医药化工、汽车配件、钢构、窑炉、造船等产业集群，力争在新材料、新能源、信息技术、节能环保等新兴产业发展上有新突破，在争创省级重点产业集群建设上有新成效。实施质量兴市战略，加强质量公共检测平台建设，深入开展大别山质量奖评选，努力形成一批名牌产品群体。

支持企业成长。完善“大企业服务直通车”机制，支持骨干企业做大做强，培植一批产销过亿元、过10亿元的企业，力争新增规模以上企业100家，产值过10亿元企业达到10家。加快企业上市步伐，加大对23家省级上市后备企业的跟踪辅导和服务。继续推进中小企业“成长工程”，落实好国家支持小微企业的各项政策措施，建立市县两级中小企业服务中心。加强企业家队伍建设，尊重爱护企业家，激发企业家的创业创新热情。

（三）加快发展现代农业

大力推进农业产业化。加大农业领域招商引资力度，充分发挥国家地理标志保护产品的品牌优势和市场潜力，发展壮大一批农产品加工龙头企业，建设一批农产品加工园区和农产品加工大县。发挥农民专业合作社作用，完善利益联结机制。加快特色板块基地建设，重点发展优质水稻、双低油菜、棉花、反季节无公害蔬菜和茶叶、板栗、花生、中药材、速生丰产林等特色农业。开展畜禽标准化示范场四级联创，加快发展奶牛、优质三元猪、肉羊肉牛、优质禽和名特优水产品。

加强农田水利等农业基础设施建设。除险加固5座中型水库和45座重点小Ⅱ型水库，基本完成13条中小河流整治任务。积极推进华阳河水系治理等项目前期工作。继续抓好大中型灌区配套设施建设，着力解决末端渠系“最后一公里”问题，促进大中型水利设施的有机衔接，提高灌溉和排涝效率。深入开展“三万”活动，全面推进“五塘整治”丰收工程。加快农村饮水安全工程和水生态环境工程建设，抓紧策划实施一批项目。

健全农业科技创新服务体系。统筹农业品种选育、农产品加工、疾病防控、农机装备等重点环节，有效整合科技资源，大力推广一批新型实用技术，积极争创农科教结合示范县。抓好科技示范户建设，着力培养一批农村新型实用人才。

加强新农村建设和扶贫开发。继续推进全省脱贫奔小康试点县、新农村建设试点乡镇、村镇建设示范工程等试点工作，加强长江生态文明示范带建设和农村环境连片整治示范工作，完成省级“宜居村庄”示范项目建设任务，推进农村社会事业建设，完善农村公共服务体系，促进农村和谐稳定。发展壮大村级集体经济，努力化解农村债务。抢抓国家重点支持革命老区和连片特困地区的重大机遇，提高扶贫标准，加大投入力度，实行扶贫开发与农村最低生活保障制度有效衔接，打好新一轮扶贫开发攻坚战。

（四）全面推进“一区两带”建设

提升市区综合实力和城市形象。全力推进临港经济区建设，着力提升市区一体开发水平，突破性地抓好园区BT项目建设和招商引资项目落户，加快黄冈高新技术产业园与武汉东湖高新区对接，力争当年完成园区基础设施投资10亿元，引进落户亿元以上项目30个，市区规模以上工业增加值增长25%。抓紧完成城市总体规划和土地利用总体规划修编，加大项目筹资、招商引资和拆迁安置工作力度，加快城东新区核心区道路、桥梁、白潭湖治理工程和社会事业配套项目建设。继续推进市区道路刷黑改造、遗爱湖综合治理保护工程以及市政设施建设。加快旧城改造、城中村和棚户区改造步伐。深入开展“城管会战”和网格化管理，全面提升城市规划建设管理水平。支持通信基础设施建设，积极打造智慧城市。

加快发展县域经济。把壮大县域经济作为实现跨越发展的重要突破口，按照长江经济带和大别山旅游经济带“两带”协调发展布局，加速推进县域工业化、城镇化和农业现代化。加快园区建设，培育特色产业，推进集群发展，着力培育一批重点产业集群和产业基地。完善县域经济发展年度综合考核评价体系，大力培育县域经济发展排头兵。

加快推进城镇化。继续实施城镇建设“211”工程和社区建设“八个一”工程，广泛开展“楚天杯”、“大别山杯”创建活动，着力提升城镇综合承载能力，培育壮大城镇支柱产业，加快城镇社会事业建设，推进交通、通信、供电、给排水等基础设施建设和网络化发展。抓紧完成重点镇总体规划修编和示范村规划编制任务，高标准做好大别山试验区村镇规划编制工作。

（五）进一步深化改革创新

深化各项体制改革。积极推进大别山试验区建设，围绕特色产业开发、基础设施建设、农民增收、社区建设、土地流转和农民合作组织建设，全方位推进改革试验，争取重大项目建设有突破性进展。深化预算管理制度改革，全面编制政府性基金预算、社会保障基金预算和国有资本经营预算，完善国库集中支付、政府采购和非税收入管理，依法加强税收征管，确保财政收支平衡。深化公共资源交易管理体制改革，加强招标投标监督管理。依法履行出资人职责，推进国有资产整合，加强国有资产监督管理。继续深化农村综合改革，巩固集体林权制度、供销社体制等改革成果。

增强自主创新能力。综合应用科技计划立项、融资服务、人才支持等多种方式，支持和培育创新型企业。以中小企业为重点，以承接科技成果转化、提高企业技术创新能力为目标，积极探索产学研深度合作的有效模式和长效机制。加快科技孵化器建设。加大人才工作力度，建立健全从引进补贴、项目支持到风险投资全过程的人才支持体系。

加快发展民营经济和外向型经济。认真落实鼓励支持民营经济发展的各项政策措施，破除制约民营经济发展的体制性障碍，引导非公有制企业加大体制、技术和管理创新。实施积极的创业政策，让一切创业者勇于创业、安心创业。大力培育外贸经营主体，加强医药化工、船舶制造、纺织服装等出口基地建设，支持重点出口企业发展。积极创造条件，优化“大通关”环境。

大力推进节能减排和环境保护。严格执行节能减排目标责任制和行政首长负责制，确保完成省政府下达的任务。引导企业开发和应用节能技术，大力推进工业、交通、建筑等重点领域节能降耗。加强污水和垃圾处理设施建设，加快重点企业环保设施升级改造，强化运营监管，坚决杜绝建而不用现象。发展循环经济，推广清洁生产，推进废物循环利用和资源综合利用。扎实开展创建省级环保模范城活动，综合治理重点水域环境污染，优先解决工业污染、噪声油烟污染、饮用水源污染等突出环境问题。广泛开展植树造林，加强森林和林地保护，构建大别山绿色生态体系。

（六）奋力推进文化强市建设

加强思想道德建设。把建设社会主义核心价值体系贯穿于文化建设全过程，努力在全社会形成统一指导思想、共同理想信念、强大精神力量和基本道德规范。加强社会公德、职业道德、家庭美德、个人品德建设，不断提升公民文明素质和城乡文明程度。

深化文化体制改革。加快经营性文化单位转企改制步伐，分类推进国有文艺院团改革。深化公益性文化事业单位改革，突出公益性，强化服务功能，深化单位内部劳动人事、收入分配、社会保障改革，提高服务水平。进一步支持湖北省黄梅戏剧院改革发展。推进文化市场综合执法改革，整合现有文化市场执法队伍。

加强公共文化服务体系建设。制定和落实名人文化与文物事业规划实施方案，下大力抓好遗爱湖公园、市博物馆、黄梅戏大剧院等标志性文化设施建设，启动黄冈名人文化园、东坡外滩、东坡遗址恢复等重点工程建设。争创“中国书法城”。深入推进重点文化惠民工程，重点抓好公共图书馆、群艺馆、博物馆和乡镇综合文化站建设。发展农村和社区公共文化事业，抓好广播电视户户通、农村数字电影放映、县乡影院建设、文化信息资源共享、农家书屋等服务项目建设。加强文物和非物质文化遗产的抢救和保护，变文化遗产为文化发展优势。广泛开展群众性文化活动，加强社区文化、企业文化、校园文化建设。加强对文化产品创作生产的规划引导和政策激励，打造一批有较高艺术水准和社会影响力的精神文化产品。

做大做强文化产业。突出黄冈特色，加强文化聚集区、文化产业园、文化产业带的规划建设，培育发展一批重点文化产业项目，打造一批可消费、可带走、可持续发展的文化产品。发展中小文化企业，加强文化产品市场和要素市场建设，完善现代文化市场体系。实施文化品牌带动和文化“走出去”战略，办好第二届大别山旅游节和第三届东坡节等重大节会活动。

（七）加强以改善民生为重点的社会建设加强教育、卫生、体育等工作。始终坚持把教育摆在优先发展的战略地位，统筹规划，加大投入，确保财政性教育经费支出占当年财政收入比例达到国家规定的要求。着力提高教育质量，增加优质教育资源供给，促进教育公平，在基本解决“上学难”的基础上着力化解“上好学难”的矛盾。加快普及学前教育，着力改善办园条件。完善中小学布局，促进义务教育均衡发展。整合职业教育资源，增强职业教育实力。支持黄冈师院等高校建设。重视特殊教育、继续教育，积极构建科学合理的教育体系。认真实施全民科技素质行动计划，着力提高公民科学素养。深化医药卫生体制改革，完善县乡村三级医疗卫生服务体系，落实基本药物制度，形成公共卫生、医疗服务、医疗保障和药品供应“四位一体”的基本医疗卫生体系。加大重大传染病防控力度。积极推进大别山（鄂东）区域医疗中心建设。实施“健康黄冈”全民行动计划。广泛开展全民健身运动，不断提高体育竞技水平。加快黄冈市体育中心建设。加快发展防震、气象等社会事业。认真做好对台、侨务、外事、档案、方志、统计、调查、保密等各项工作。

健全基本公共服务体系。实施更加积极的就业政策，多渠道开发就业岗位，完善公共就业服务体系，以创业带动就业，以培训促进就业，以援助扶持就业，以维权保障就业。重点做好高校毕业生、农村转移劳动力、就业困难人员和退役军人的就业工作。进一步完善社会保障体系，继续扩大社会保险覆盖面，实现城乡居民社会养老保险全覆盖。逐步提高城镇职工、城镇居民基本医疗保险和新型农村合作医疗保障水平。完善各类社会保险跨区域转移接续政策，基本实现医疗、失业、工伤、生育社会保险市级统筹。建立健全城乡低保应保尽保动态管理机制。健全困难群众临时救助制度，建立社会救助、保障标准与物价上涨挂钩联动机制。健全住房公积金制度。加强农村福利院建设。积极发展社会福利事业和慈善事业。重视发展社会养老事业，切实关心空巢老人和留守妇女儿童。加快残疾人社会保障和服务体系建设。严格控制政策外生育，综合治理出生人口性别比，统筹解决人口问题，不断提高人口素质。

加强和创新社会管理。完善城乡社会管理体系，建立健全公共服务保障、公共诉求管理、重点人群管理、社会组织管理、社会矛盾化解、社会稳定风险评估等工作机制，提高应急管理能力。加强城市社区管理网格化信息平台建设，做好农村流动人口、留守人员的服务管理工作，深入推进法治黄冈、平安黄冈建设，加强社会管理综合治理，健全打防控一体化的社会治安防控体系，加大“打黑除恶”、反抢反盗等严打工作力度，切实提高人民群众的安全感和满意度。强化安全发展理念，健全安全生产责任体系，标本兼治、综合治理，坚决遏制重特大事故发生。切实做好消防工作。进一步强化全民国防意识，加强民兵预备役建设，提升国防动员能力，支持驻黄部队建设，抓好“双拥”工作。加强基层民主政治建设，不断完善村民、居民自治。继续做好援疆和移民工作。依法管理宗教事务，巩固民族团结。

实施惠民工程。进一步加大投入、拓展内容、完善措施，着力办好10件惠民利民实事：（1）城镇新增就业4万人，帮助农村富余劳动力转移就业5万人。（2）建设保障性住房30万平方米，改造农村危房1万户。（3）再解决农村10万人饮水安全问题；实施清洁能源入户工程6万户；完成塘堰整治4万口。（4）继续实施农村环境连片整治示范工程，建成349个农村生态文明示范村。（5）完成“12315”消费维权服务站规范化建设100家。（6）为260名0到6岁贫困残疾儿童实行抢救性康复；实施贫困精神病患者救助2000名。（7）在100个行政村建设农民体育健身工程。（8）继续为困难群众和农民工提供法律援助。（9）建成义务教育阶段标准化学校200所，新建、改建、扩建乡镇公办幼儿园40所；加快调整优化教育布局，启动市区5所中小学新建、迁建工程。（10）市区新建和改造标准化集贸市场10个。

（八）切实加强政府自身建设

以建设法治政府、服务政府、责任政府和效能政府为目标，依法行政，从严治政，廉洁从政，着力提高科学发展的能力和水平。

增强依法行政的自觉性。坚决贯彻落实市委各项决策，自觉接受市人大法律监督、工作监督和市政协民主监督，认真办理人大议案、代表建议和政协提案，广泛听取各民主党派、工商联和无党派人士的意见建议。规范执法行为，强化执法监督，加强行政复议工作。健全信访机制，推进“六五”普法。推行电子政务，加强电子监察，深化政务公开，打造阳光政府。

增强政务服务的主动性。加强调查研究，广泛了解民情民意，问政于民、问计于民、问需于民，强化民主科学决策。大力治庸问责，凡是定下来的事情立即办、马上办，抢时间、保质效，事不过夜、一抓到底。转变政风行风，下决心精简会议和文件，围绕提高审批效率，减程序、减时限、减费用，再造审批流程，完善市县乡村四级行政服务体系，努力打造行政审批流程最优、环节最少、时限最短、服务最佳的政务服务环境。

增强廉洁从政的坚定性。全面落实“一岗双责”，深化“十个全覆盖”工作，加大源头治腐力度，切实解决损害群众利益的突出问题。健全政府采购、资产转让、项目招投标、土地招拍挂等管理制度，加强重点领域、关键岗位廉政风险预警防控。继续抓好领导干部经济责任审计，加强专项资金使用、政府投资项目的审计和监察，严肃查处违法违纪案件，以反腐倡廉的实效取信于民。

各位代表！完成未来五年和今年的目标任务，实现对人民的庄严承诺，使命崇高，责任重大。让我们更加紧密地团结在以胡锦涛同志为总书记的党中央周围，在市委的正确领导下，深入贯彻落实科学发展观，以更加振奋的精神、更加开阔的视野、更加务实的作风，为推动经济社会跨越发展、建设富裕文明新黄冈而努力奋斗！
